# Synchronous lung and multiple soft tissue metastases developed from osteosarcoma of tibia: a rare case report and genetic profile analysis

**DOI:** 10.1186/s12891-022-05020-6

**Published:** 2022-01-20

**Authors:** Chuanxi Zheng, Yitian Wang, Yi Luo, Zongguo Pang, Yong Zhou, Li Min, Chongqi Tu

**Affiliations:** 1 Department of Orthopedics, Orthopedics Research Institute, West China Hospital, Sichuan University, Guoxue Road No. 37, Chengdu, Sichuan 610041 People’s Republic of China; 2 Department of Pathology, West China Hospital, Sichuan University, Guoxue Road No. 37, Chengdu, Sichuan 610041 People’s Republic of China

**Keywords:** Osteosarcoma, Extrapulmonary, Metastasis, Germline mutation, ALK

## Abstract

**Background:**

Osteosarcoma is the most common primary malignant bone tumor with a highly metastatic propensity in children and young adolescents. The majority of metastases develope in the lung, while metastases to the extrapulmonary locations have rarely been discussed, especially in skeletal muscle.

**Case presentation:**

We reported a young patient with pathologically diagnosed osteosarcoma of the right tibia who was initially treated with standard chemotherapy and complete surgical resection. However, pulmonary metastases and multiple soft tissue masses in skeletal muscle developed four years after the index surgical resection. Subsequently, a targeted next-generation sequencing assay based on an 806 oncogenes and tumor suppressor genes panel was performed to analyze genetic alterations in this patient with rare metastatic pattern. The genetic analysis revealed canonical somatic mutations of *RB1* and germline variants of *ALK* (c.862 T > C), *BLM* (c.1021C > T), *PTCH1* (c.152_154del), *MSH2* (c.14C > A), *RAD51C* (c.635G > A). Using silico prediction programs, the germline variants of the *MSH2* and *RAD51C* were predicted as “Possibly Damaging” by Polymorphism Phenotyping v2 (PolyPhen-2) and “Tolerated” by Sorting Intolerant from Tolerant (SIFT); *BLM* was classified as “Tolerated”, while the germline variant of *ALK* was predicted to be pathogenic by both PolyPhen-2 and SIFT.

**Conclusions:**

Osteosarcoma with extrapulmonary metastases is rare, especially located in the skeletal muscle, which predicts a worse clinical outcome compared with lung-only metastases. The several novel variants of *ALK, BLM, PTCH1* in this patient might expand the mutational spectrums of the osteosarcoma. All the results may contribute to a better understanding of the clinical course and genetic characteristics of osteosarcoma patients with metastasis.

## Background

Osteosarcoma is the most common primary malignant bone tumor with a highly metastatic propensity in children and young adolescents [1]. The advent of neoadjuvant chemotherapy has improved the 5-year overall survival rate of osteosarcoma from less than 20% to over 70% [2]. However, metastasis and relapse are the common adverse predictor factors for patients with osteosarcoma. At the time of diagnosis, approximately 15% to 20% of patients have clinically detectable metastases and the 5-year overall survival rate of those patients is usually less than 30% [3–7]. The majority of osteosarcoma metastases occurre in the lung, while metastases to the extrapulmonary locations have been rarely reported, such as breast, abdominal viscera, chest wall, heart, penis, brain, subcutaneous tissue, skeletal muscle [8–15]. Among these extrapulmonary involvement, skeletal muscle metastases are extremity rare which account for only 1.6% of patients with metastases from osteosarcoma and there are only several relevant case reports that have been published to date [16].

The clinical outcome of the patients with extrapulmonary metastases is much worse than that of the patients with lung-only metastases [16]. For patients with resectable lung metastases, the combination of aggressive chemotherapy with metastasectomy could effectively achieve disease control and improve the prognosis, whereas systematic chemotherapy seems invalid for patients who have developed extrapulmonary metastases [8, 16]. The dismal outcome of patients with extrapulmonary metastases emphasized the importance of understanding the underlying molecular mechanisms and specific genetic profile of this metastatic pattern, which so far remain unknown. To our knowledge, none of the published cases had investigated the genetic profile of the patient with skeletal muscle metastasis from osteosarcoma. Here, we reported a case of patient with osteosarcoma who developed skeletal metastases four years after the initial treatment. Synchronously, we investigated the molecular genetic profile of tumors sample in an effort to better understand the clinical course and genetic characteristics of osteosarcoma patients with soft tissue metastasis.

## Case presentation

**Fig. 1:**
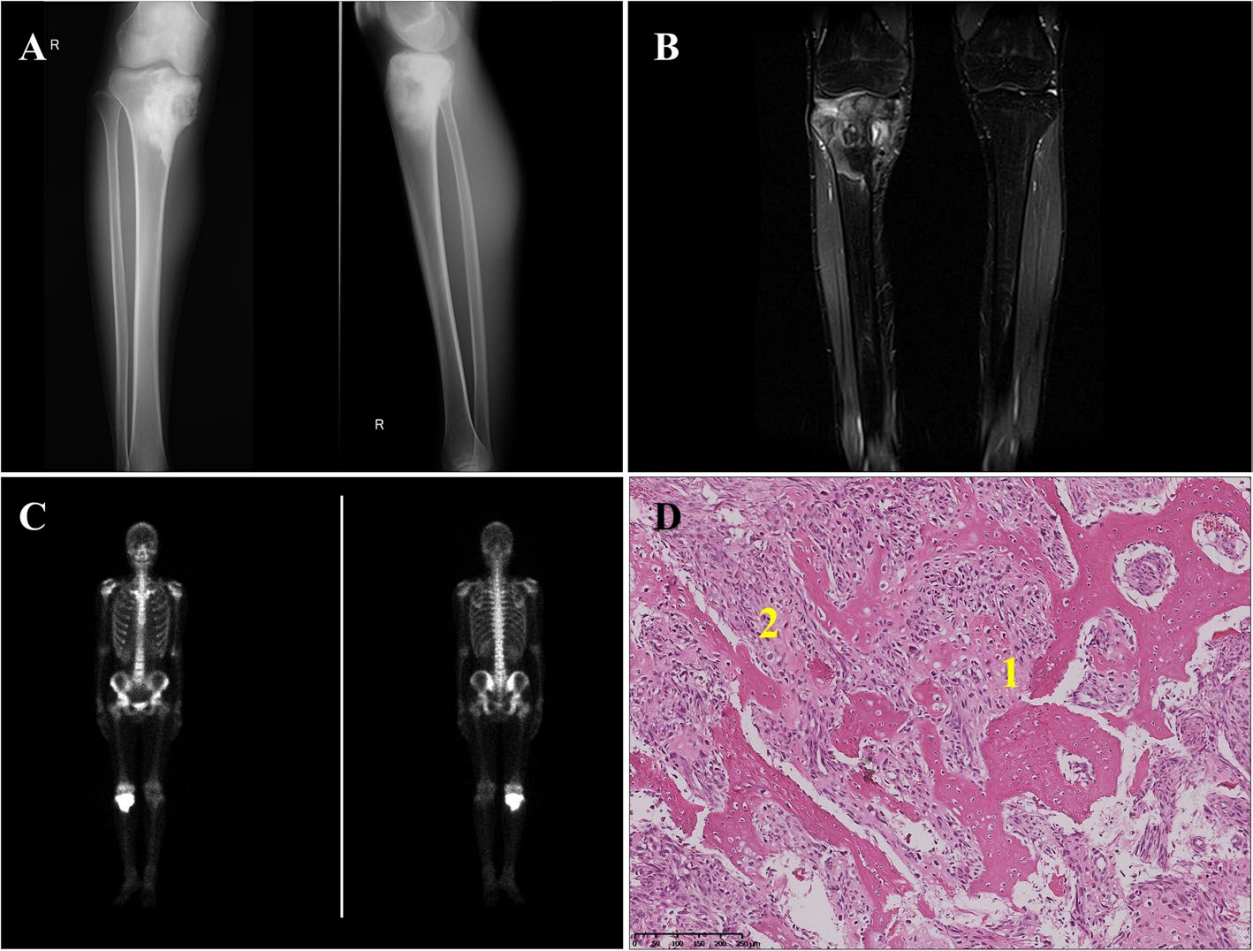
The radiological and histological findings of the primary osteosarcoma in the right tibia. Anteroposterior and lateral radiograph showed (**A**) an ill-defined osteolytic lesion with osteoid matrix involving the proximal metaphysis of the right tibia. Coronal fat-saturated T2-weighted MRI image (**B**) demonstrated an intramedullary lesion of heterogeneously high signal intensity with cortical breaching and soft tissue extension. Bone scintigraphy (**C**) presented diffusely increased activity in the right proximal tibia. **(D)** Pathological examination of the biopsy specimen revealed severe cytological atypia and regions of the eosinophilic chondroid matrix with chondroblastoma-like neoplastic cells **(1)** and neoplastic bone production **(2)**

A 17-year-old young patient with a one-month history of dull pain and limited motion of the right knee was admitted to the orthopedic department of West China Hospital in July 2016. The patient reported no relevant oncological family history. Physical examination revealed tenderness in the medial metaphysis of the right tibia and limited mobility of the right knee. The anteroposterior and lateral radiograph showed an ill-defined osteolytic lesion with osteoid matrix involving the proximal metaphysis of the right tibia (Fig. 1A). Magnetic resonance imaging (MRI) revealed an intramedullary lesion of heterogeneously high signal intensity with cortical breaching and soft tissue extension in the proximal metaphysis of the right tibia (Fig. 1B). Computed tomography (CT) scan of the chest did not find the distant metastasis and bone scintigraphy only presented diffusely increased activity in the right proximal tibia (Fig. 1C). Subsequently, an incisional biopsy of lesion in the right tibia was performed. The pathological findings revealed severe cytological atypia and regions of the eosinophilic chondroid matrix with chondroblastoma-like neoplastic cells and osteoid formation, which confirmed the diagnosis of chondroblastoma-like osteosarcoma (Fig. 1D). After three courses of neoadjuvant chemotherapy consisting of doxorubicin (30 mg/m^2^ on days 1 to 2) and cisplatin (120 mg/m^2^ on day 1), the patient underwent segmental resection of the lesion with prosthetic reconstruction. While the histological response rate of the resected specimen was less than 50% of the entire lesion, indicating the patient might be refractory to the first-line neoadjuvant chemotherapy. Following surgery, the second-line adjuvant chemotherapy consisting of doxorubicin (30 mg/m^2^ on days 1 to 2), cisplatin (120 mg/m^2^ on day 1) and ifosfamide (3 g/m^2^ on days 1 to 4) were administrated. However, it was discontinued after two courses of treatment due to patient refusal and substituted by the anti-angiogenesis therapy with apatinib, an oral tyrosine kinase inhibitor. Initially, apatinib was administrated at an initial dose of 500 mg once daily with informed consent for off-label use. Due to the drug-related toxicity of the wound dehiscence and hair hypopigmentation, the patient discontinued apatinib treatment after two months by his own decision and then lost to the periodic clinical assessment in the subsequent follow-up.

**Fig. 2:**
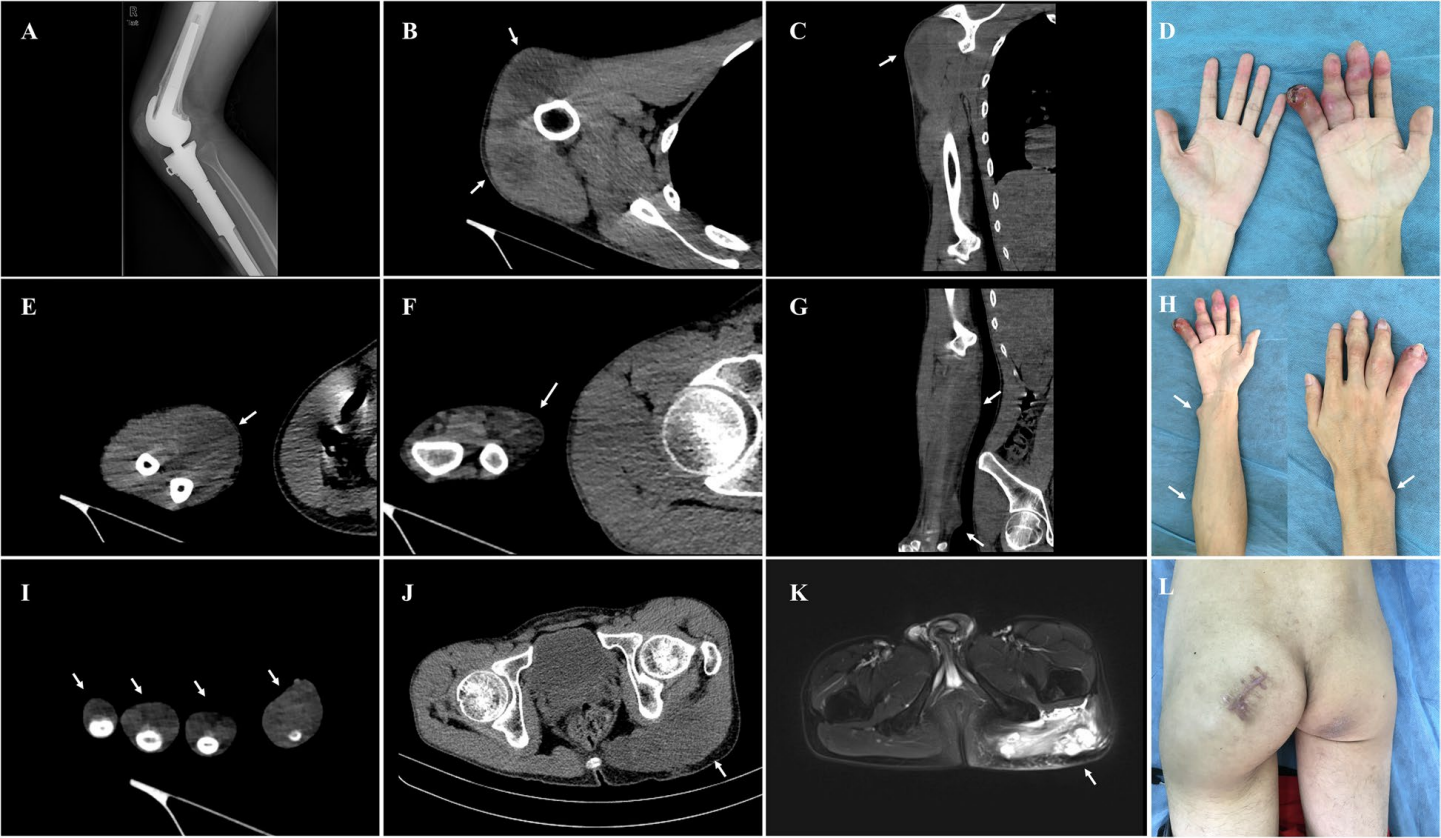
The radiological findings of the metastatic lesion from osteosarcoma. The radiograph showed no local recurrence around the right knee (**A**). The CT scan of whole body demonstrated multiple soft masses without bone destruction in the right deltoid muscle (**B, C**), right forearm (**E, F, G, H**), the 2, 3, 4, 5 digits of the right hand (**D**, **I**) and left gluteal maximus muscle (**J, L**). Corresponding axial fat-saturated T2-weighted MRI image of the pelvis (**K**) depicted a soft tissue mass with high signal intensity in the gluteal maximus muscle

**Fig. 3:**
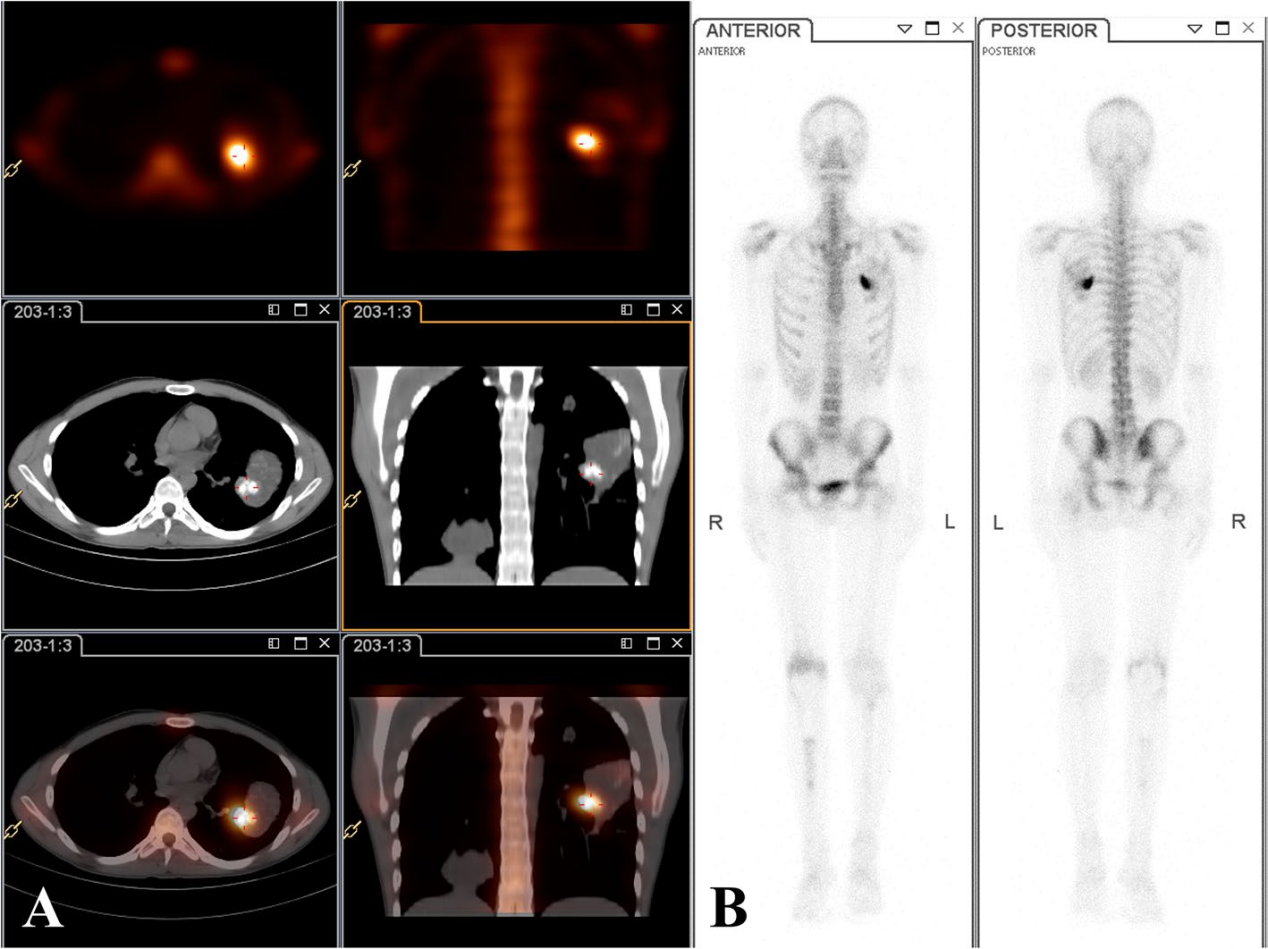
Single-photon emission computed tomography of the lung metastatic lesions in the bilateral lung. Single-photon emission computed tomography of the chest showed multiple pulmonary metastatic lesions in which the largest nodules in the left upper lobe with internal calcification (**A**). Bone scintigraphy showed increased uptake in the lesion of the left lung corresponding to the metastatic lesion (**B**)

Unfortunately, the patient presented to our orthopedic clinic again with a complaint of polypnea and multiple soft tissue masses in the right hand and left buttock in April 2020. Physical examination revealed multiple firm, palpable, tender masses in the right shoulder, forearm, left buttock, and the 2, 3, 4, 5 digits of the right hand, without any local recurrence around the right knee (Fig. 2A, D, H, L). The CT scan of whole body demonstrated multiple soft masses without bone destruction in the right deltoid muscle, right forearm, left gluteal maximus muscle, and the 2, 3, 4, 5 digits of the right hand (Fig. 2B, C, E, F, G, I, J). Single-photon emission computed tomography revealed multiple pulmonary metastatic lesions in the bilateral lung in which the largest nodule measuring 7.6 cm × 4.2 cm × 5.0 cm was located in the left upper lobe with internal calcification (Fig. 3A). Bone scintigraphy indicated increased activity lesions in the sites corresponding to the nodule in the left lung (Fig. 3B). An incisional biopsy of lesion in the left gluteal maximus muscle was conducted, the pathological findings showed atypical cell proliferation and osteoid formation consistent with the microscopic presence of osteosarcoma. Based on the medical history, radiological and pathological findings, the diagnosis of multiple metastases from osteosarcoma in the bilateral lung and skeletal muscles was established. Nevertheless, the subsequent Eastern Cooperative Oncology Group (ECOG) performance score of the patient was 3 indicating a poor tolerance for palliative chemotherapy and his family was also counseled on the poor prognosis of the disease. Eventually, the patient and his family were reluctant to undergo palliative chemotherapy and decided to pursue another further targeted treatment.

## Genomic analysis

To further investigate genetic alterations of this rare case, somatic tumor testing on soft tissue metastasis was performed. Tumor DNA was extracted from the fresh biopsies of tumor cells (frozen in liquid nitrogen, and stored at -80℃) using DNA Extraction Reagent Kit (TIANGEN, Beijing, China). Meanwhile, genomic DNA was extracted from whole peripheral blood using standard phenol–chloroform extraction protocol to aid in determining germline variants. The cancer-specific mutation status was evaluated with the next-generation DNA sequencing for 806 oncogenes and tumor suppressor genes from the cancer panel (illumina NovaSeq 6000 System, United States).

**Fig. 4:**
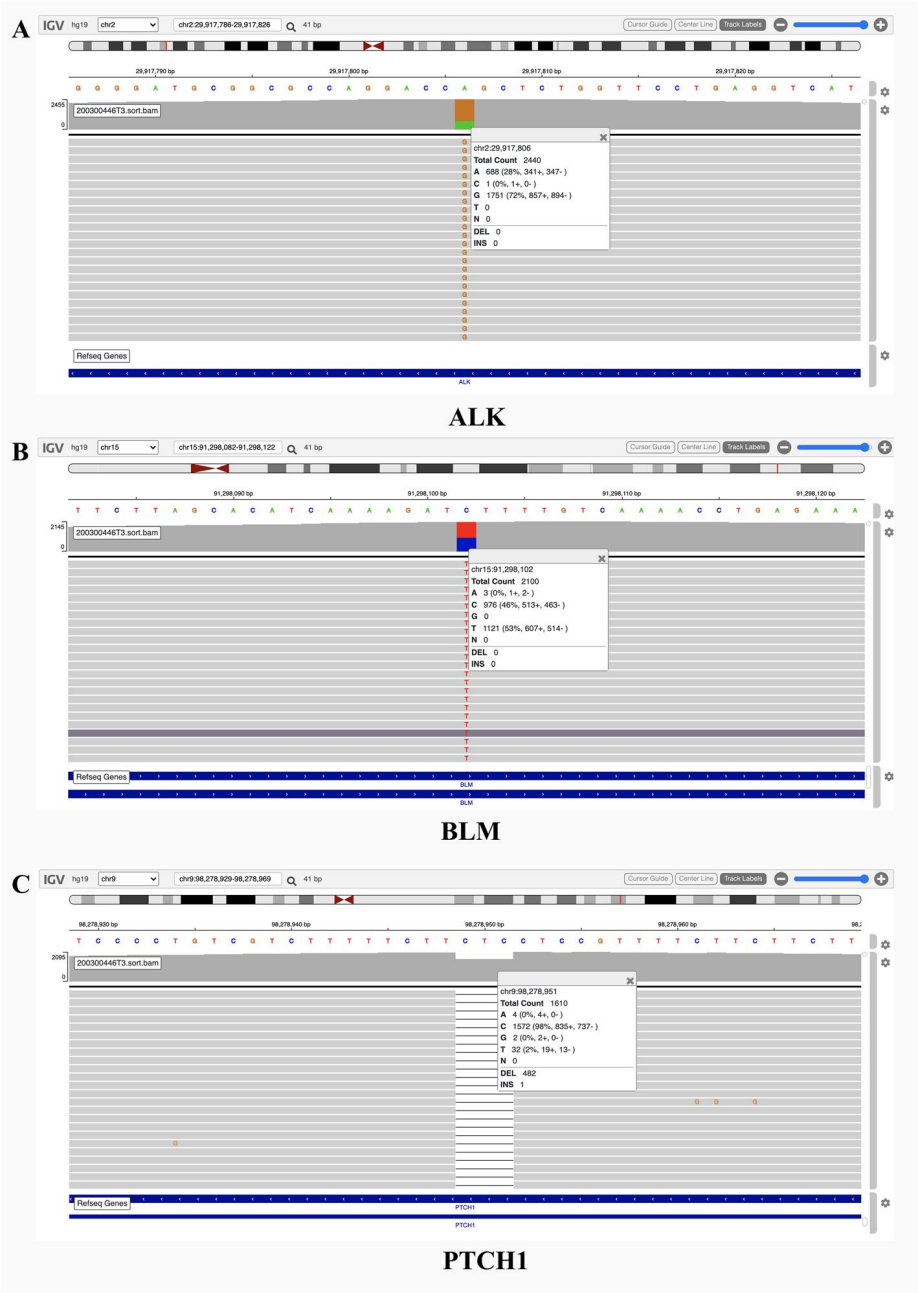
The novel germline variants were identified in the patient. Sequencing reads of *ALK* (**A**), *BLM* (**B**), *PTCH1*(**C**) were shown by using the Integrative Genomic Viewer browser

Sequencing analysis of the sample acquired from the gluteal metastasis revealed a microsatellite-stable tumor with a low tumor mutational burden (1.02 mut/MB) and immunohistochemical staining was negative for PD-L1 expression. Genes with alternation in the tumor sample were summarized in Table 1. Panel sequencing analysis of the 806 genes identified previously reported somatic mutation in *RB1*, exon 21 c.2211+1G>C (COSM7154357, COSM7154356), as well as heterozygous germline mutation in *MSH2* exon1, c.14C>A/ p.P5Q (dbSNP:rs148098584) and *RAD51C*, exon4, c.635G>A/ p.R212H (dbSNP:rs200857129). Importantly, the therapeutic implication of the somatic mutation in *RB1* indicated the patient might be sensitive to mTOR inhibitors. Above the aforementioned variants, several rare germline variants of *ALK* (exon3, c.862T>C/p.W288R), *BLM* (exon5, c.1021C>T/p.L341F), *PTCH1* (exon1, c.152_154del/p.51_52del) were also identified in this patient (Fig. 4). These variants were further assessed for possible pathogenicity and the effects on protein function by using the bioinformatic programs, including Sorting Intolerant from Tolerant (SIFT), Polymorphism Phenotyping v2 (PolyPhen-2). Among the genetic alterations, germline variants of the *PTCH1, MSH2* and *RAD51C* were predicted as “Possibly Damaging” by Polyphen-2 and “Tolerated” by SIFT; *BLM* was classified as “Tolerated”, while the germline variant of *ALK* was predicted to be damaging by both PolyPhen-2 and SIFT. However, all five germline mutations in the tumor sample were classified as variants of uncertain significance (VUS) in ClinVar (http://www.ncbi.nlm.nih.gov/clinvar). Based on the therapeutic implication of somatic mutation in the *RB1* which might be sensitive to the targeted therapy of mTOR inhibitor, and everolimus was orally administrated with a dose of 10mg daily. However, the disease still progressed rapidly and the patient died from the complication of lung metastasis 2 months later.

**Table 1:** Genes with alteration in the sample from the skeletal muscle metastasis of the patient

Gene	Location	Nucleotide change	Amino acid change	Gene ID in databases	Mutation pattern	SIFT function	Polyphen-2 function	Therapeutic implication
** Somatic mutations**								
RB1	exon21	c.2211 + 1G > C	NA	COSM7154357	Splicing	NA	NA	mTOR inhibitor
				COSM7154356				
** Germline mutation**								
ALK	exon3	c.862 T > C	p.W288R	NA	Heterozygosis	Damaging	Damaging	VUS
BLM	exon5	c.1021C > T	p.L341F	NA	Heterozygosis	Tolerated	Benign	VUS
PTCH1	exon1	c.152_154del	p.51_52del	NA	Heterozygosis	Tolerated	Damaging	VUS
MSH2	exon1	c.14C > A	p.P5Q	rs56170584	Heterozygosis	Tolerated	Damaging	VUS
RAD51C	exon4	c.635G > A	p.R212H	rs200857129	Heterozygosis	Tolerated	Damaging	VUS

*NA* not achieved, *VUS* variant of uncertain significance

## Discussion and Conclusion

Due to the resistant mechanism of skeletal muscle to metastatic deposits, metastases to soft tissue from primary malignancies are rare [17]. In osteosarcoma, skeletal muscle metastases are extremity rare accounting for about 1.6% of patients who developed metastatic lesions from primary sites [16]. Since the introduction of chemotherapy, the metastatic pattern of osteosarcoma has altered with a higher incidence of extrapulmonary metastases in patients who underwent adjuvant chemotherapy, with or without concurrent pulmonary metastases [18]. While the clinical features, therapeutic strategies and prognosis between the patients with lung-only and extrapulmonary lesions are markedly distinct. The latter metastatic pattern usually indicates the probability of disease dissemination, a poor histologic response to preoperative chemotherapy and difficulty in complete resection of metastatic lesions, which are associated with worse clinical outcomes [19, 20]. The median overall survival was 26.0 months for those with lung-only metastasis, but only 12.7 months for patients who developed extrapulmonary metastasis [16]. In the present case, the patient was resistant to the first-line chemotherapy with a histological response rate of less than 90% that was similar to the previously reported cases with skeletal muscle metastases [8, 20]. As the second-line therapy, additional ifosfamide chemotherapy was administrated, whereas it seemed insufficient to control disease progression. Although oral tyrosine kinase inhibitors apatinib had been demonstrated effective in the management of advanced osteosarcoma after the failure of multimodal therapy [21], the patient terminated apatinib treatment within two months due to the intolerable adverts side effects. Consequently, the patient developed multiple metastases in the bilateral lung and skeletal muscles. In this setting, the intensive chemotherapy combined with aggressive metastasectomy was not feasible leading to a dismal clinical prognosis.

Unlike other solid tumors, osteosarcoma exhibits chromosomal instability characterized by intra-tumoral and inter-tumoral heterogeneity with a higher mutation rate [22]. Several cancer predisposition syndromes have been established to be associated with osteosarcoma, including Li-Fraumeni syndrome and Diamond-Blackfan anemia, Rothmund-Thomson syndrome, Baller-Gerold syndrome, RAPADILINO syndrome, Werner syndrome, Bloom syndrome, ATR-X syndrome [23–28]. In addition to the syndrome-related osteosarcoma, increasing pathogenic germline mutation has been identified in osteosarcoma individuals with use of the DNA sequencing which may contribute to the complex underlying mechanism of osteosarcoma development. A sequencing study of 1120 cases showed 7/39 osteosarcoma patients harboring pathogenic and likely pathogenic variants in *TP53, RB1, APC, MSH2,* and *PALB2* [29]. Another targeted exon sequencing study involving 1162 patients with sarcoma revealed that more than 50% of all patients carried pathogenic variants in *TP53, BRCA2, ATM, ATR*, and *ERCC2* [30]. More recently, an emerging study investigating the germline genetic architecture of 1244 patients with osteosarcoma demonstrated that 28% of patients possessed pathogenic or likely pathogenic cancer-susceptibility genes variants and identified new candidate genes including *CDKN2A, MEN1, VHL, POT1, APC, MSH2* and *ATRX* [31]. In the present case, we likewise identified the heterozygous mutation in *MSH2, RAD51C* as previously reported in osteosarcoma [31]. Furthermore, we observed several novel germline variants of *ALK* (c.862 T > C), *BLM* (c.1021C > T), *PTCH1* (c.152_154del) in this patient. However, only the germline variant of *ALK* was predicted to be pathogenic by using in silico prediction programs.

Aberrations in the oncogene *ALK* have emerged as potentially relevant biomarkers and therapeutic targets in several solid tumors, including neuroblastoma, inflammatory myofibroblastic tumor, and non-small-cell lung cancer [32]. Moreover, the *ALK* has been found to be rearranged, mutated, or amplified in Ewing sarcoma and rhabdomyosarcoma [32–34]. The immunopositivity expression of *ALK* protein was also observed in 30% ~ 40% of patients with soft tissue sarcoma (including osteosarcoma) and correlated with a poor clinical course [35, 36]. In the synovial sarcoma cell lines, the *ALK* variant with a large extracellular domain deletion encoding by the absence of exons 2–17 and exon1- exon18 splicing was identified as a novel driver gene [37]. Subsequent functional analysis demonstrated this alteration activated multiple proliferative and survival pathways, resulting in a remarkable dependency on *ALK* for tumor cells growth both in vitro and in vivo [37]. Moreover, ALK-positive patients harboring *ALK* rearrangement in both primary synovial sarcoma and metastatic lesions further validated the role of *ALK* in synovial sarcoma and indicated *ALK* aberration may be required for metastatic progression [37]. Recently, a novel *ALK* transcript initiated from a de novo alternative transcription initiation (ATI) site in *ALK* intron 19, ALK^ATI^, was frequently detected in soft tissue sarcoma [36]. In vitro and *vivo*, ALK^ATI^ drove tumorigenesis and enhanced cancer stem cell-like properties through interacting with *c-Myc* and promoting the binding of *c-Myc* to the *ABCG2* promoter [36]. So far, few studies have systematically investigated the role of *ALK* in osteosarcoma, although somatic *ALK* loss (c.*60_*61insCAAT) and mutation (p.K911T and p.A585T) have been reported in human osteosarcoma samples [38, 39]. However, these aforementioned results implied *ALK* alteration might play an important role in the tumor progression of osteosarcoma.

Importantly, *ALK* mutation or rearrangement has been shown to lead to resistance of tumor cells to both radiotherapy and chemotherapy, but sensitiveness to *ALK* inhibitors [36, 37]. Several tyrosine kinase inhibitors targeting *ALK* were recently validated in clinical trials, such as brigatinib, crizotinib and ceritinib which have been approved by the Food and Drug Administration (FDA) for the management of inflammatory myofibroblastic tumors with *ALK* translocation [40, 41]. Mosse and colleagues have reported 86% of patients with ALK-fusion inflammatory myofibroblastic tumors responded to crizotinib and 36% of patients achieved a complete response [42]. Moreover, Jiao et al. reported a patient with metastatic low-grade sarcoma carrying *CARS-ALK* fusion who was dramatically responded to multiple ALK tyrosine kinase inhibitors (crizotinib and alectinib) after treatment failure of the first-line chemotherapy and successfully survived for more than 5 years with a durable response [43]. In this context, we speculated whether the present patient with mutation of *ALK* could benefit from ALK inhibitors treatment after failure of chemotherapy, which might halt the disease progression or improve the survival of the patient to some extent. However, the efficacy and potential benefit of ALK inhibitors in the adjuvant setting for osteosarcoma require further confirmation in future studies.

In conclusion, osteosarcoma with extrapulmonary metastases is rare, especially in the skeletal muscle, which predicts a worse clinical outcome compared with lung-only metastases. Additionally, several novel mutations have been identified in this study which would enrich the mutational spectrums of osteosarcoma. More information about the biological relevance of these mutations will be helpful to shed new light on the therapeutic targets for this refectory disease.

## Data Availability

All data used or analyzed during this study are included in this published article.
